# Digitisation and Sharing of Collections: Museum Practices and Copyright During the COVID-19 Pandemic

**DOI:** 10.1007/s11196-023-09986-x

**Published:** 2023-04-20

**Authors:** Mateusz Klinowski, Karolina Szafarowicz

**Affiliations:** Jagiellonian University, Kraków, Poland

**Keywords:** Virtual museum, Copyright, DSM Directive, Digitisation, COVID-19

## Abstract

This article concerns the conflict between copyright and museums’ digitisation and online sharing of collections. This issue has recently become particularly important in connection with the COVID-19 pandemic. The authors outline the concept of a virtual museum and present the most important copyright provisions in EU law that may create obstacles for cultural institutions in realising virtual counterparts. To perceive copyright as the main obstacle in the process of digitisation and online sharing of collections is not unusual. Hence, the article briefly presents legal framework of the European copyright applicable to such situations. The authors argue that although copyright offers a range of possibilities for museums interested in digitising their collections, at the same time it is responsible for a *chilling effect*, resulting in fear of potential infringement and liability. The authors conclude that the EU’s development of new legislation, coinciding with the need for digitisation and online sharing of cultural heritage caused by the pandemic, has favoured public interest at the expense of creators’ rights, but still lacks satisfactory legal tools for effectively allowing cultural institutions to digitise and share their collections.

## Introduction

This article addresses the problem of copyright restrictions on digitisation and the online sharing of museum collections. Although this issue has been already a subject of theoretical consideration for some time, it has recently gained particular practical importance due to the COVID-19 pandemic. Cultural institutions were among the first ‘victims’ of the pandemic, forced to stay closed due to lockdowns and restrictions on social life. In response, they tried to move a large portion of their activities online. After outlining how the pandemic has affected the activities of museums and reviving the idea of virtual museums, the authors describe how museums must address copyright issues, focusing specifically on different ways copyright can create obstacles to fulfilling museums’ social mission. The authors also comment on some of the provisions of the Directive (EU) 2019/790 of the European Parliament and of the Council of 17 April 2019 on copyright and related rights in the Digital Single Market (hereinafter: DSM Directive), considering whether and how it may affect the activities of museums. The article concludes by reflecting on possible developments in copyright and trends in cultural practices evolving towards favouring the public interest at the expense of creators’ rights.

The research hypothesis is that although the pandemic has significantly accelerated the development of the idea of the virtual museum, copyright remains a serious problem despite significant attempts to mitigate it within EU law. Specifically, the DSM Directive has not been entirely successful as a tool to facilitate the functioning of cultural institutions and has not kept up with the dynamic changes currently taking place in the way cultural institutions function.

## Bringing the Sector of Cultural Institutions Online

### COVID-19 Pandemic

The creative and cultural sectors were among those most damaged financially by the COVID-19 pandemic. The pandemic was a global outbreak of infectious disease caused by the respiratory virus SARS-CoV-2. During the initial phase of the pandemic, which the WHO officially declared on 11 March 2020, European governments implemented unprecedented (at least in the post-World War II era), stiff limitations on social activities and citizens’ mobility in order to reduce person-to-person contact. These measures were motivated by the difficult situation in Italy, especially in Lombardy, where unrestricted early transmission of the virus led to high morbidity and mortality among the local population. In the absence of vaccines and effective therapies, reduction of social activity and stay-at-home orders seemed to be the only reasonable solution to the acute infection. Different countries around the world introduced ‘national quarantines’ varying in duration and severity. Mandatory lockdowns of restaurants, pubs and schools as well as restrictions on public transportation, especially international flights, were the most common measures. The reduction of social mobility, although enforced by governments, was actually mainly driven by individual choices motivated by the fear of infection. In fact, one study demonstrates that consumer traffic in the USA at the beginning of the pandemic fell by 60%, but legal restrictions were responsible for only 7% of this drop [1].

Despite having much less importance in comparison to commerce in generating social contact and individual mobility, cultural institutions like libraries and museums were also struck by lockdowns, resulting in total or part-time closures, staff reduction, and reduction in the scope of cultural and educational programs and exhibitions. For example, according to UNESCO data, over 70% of sites ranked as World Heritage properties were closed at the beginning of the pandemic [2]. The survey conducted by the International Council of Museums (ICOM) indicated that in the first phase of the pandemic (April–May 2020), almost 95% of museums were closed worldwide [3] and suffered significant financial deterioration as a result. A study conducted in Poland demonstrated that 72.5% of surveyed cultural institutions experienced a decrease in revenues of over 50% [4]. The study also indicated that digitisation was a common response to the crisis for libraries and museums. Other entities such as theatres as well as independent artists also moved to the Internet in search of audiences and profits.

The search for an audience, while usually overshadowed by financial aspects of running of cultural institutions, constitutes a valid motive for digitisation during lockdowns. The question of usefulness, of feeling ‘important or needed’ is usually ignored in scholarly discussions on the meaning of the pandemic for the creative, cultural and entertainment sectors. Nonetheless, usefulness to society seems to be part of the reason for creating cultural institutions in the first place and for maintaining their existence. Hence, although digitisation is usually presented in the literature as a means of regaining lost revenue, its drivers are much more meaningful and are deeply rooted in the very nature of cultural activity.

Because institutions in the cultural sector of economy are perceived and usually defined in terms of their mission based on education, research, and leisure and are sometimes (particularly in the case of museums) primarily non-profit organisations,^1^ during the pandemic these institutions sought different methods for retaining their functions and generally turned to the Internet. The Internet became important for the whole cultural and creative industry (CCI), also because of growing demand from the public. People – often locked in their homes and unable to go outside even to work – turned to cultural activities they did not have time for before. By necessity, most of that activity was facilitated by different online services such as streaming platforms. However, even more traditional cultural institutions experienced a sudden growth in their services. For example, a UK report revealed a 146% increase in borrowed e-books and a 27% increase in membership to access digital resources of British libraries in the first four months of the pandemic [5]. All these drivers and circumstances play an important role in explaining and justifying the shape of regulations concerning virtual museums both during the time of the pandemic and beyond.

### The Concept of Virtual Museums and its Development

The concept of the virtual museum is not new. In fact, the idea of virtual museums already existed before the appearance of the Internet [6]. André Malraux, a French writer and art historian, is often indicated as the originator of the concept. In his essay “Le Musée imaginaire” dated 1947, he “developed the idea of an imaginary museum that would bring together more works than even the greatest of museums could ever assemble within its walls” [6], which later came to be known as ‘the museum without walls’ [7]. Not long afterwards, in the 1960s, discussions emerged about various technical solutions that could be used by museums for research, sharing of information or learning purposes [6]. However, only recent technological developments such as hypertext, interactive multimedia, digitisation and the Internet have made it possible for museums to realise these visions [6, 8]. Currently artificial intelligence (AI) is even used to create art, and 3D scanning in connection with the VR googles technology or even mobile phone cameras (augmented reality) mark an uncharted territory for virtual museum creations [9]. Although these particular issues are outside of the scope of our paper, deserving a separate study due to their complexity and novelty, copyright considerations regarding more “traditional” modes of digitalization of museum objects will be applicable to them as long as there are reproductions to be made.

Despite the fact that the concept has been present in the literature over the last 30 years, there is no unanimous definition of a virtual museum, and terms including ‘online museum’ and ‘digital museum’ are often used interchangeably [6]. A relatively broad definition can be found in the Encyclopaedia Britannica, according to which a virtual museum is

“a collection of digitally recorded images, sound files, text documents, and other data of historical, scientific, or cultural interest that are accessed through electronic media. A virtual museum does not house actual objects and therefore lacks the permanence and unique qualities of a museum in the institutional definition of the term. In fact, most virtual museums are sponsored by institutional museums and are directly dependent upon their existing collections” [10].

Another definition was proposed by the Virtual Multimodal Museum (ViMM),^2^ according to which a virtual museum is “a digital entity that draws on the characteristics of a museum, in order to complement, enhance, or augment the museum through personalization, interactivity, user experience and richness of content”. However, the problematic nature of this definition lies in the fact that, firstly, it refers to the definition of a traditional museum, which also varies in formulation and is subject to change [11, 12]; and, secondly, it focuses on the subservient role of the virtual museum rather than allowing it to exist independently. Others, making a major simplification, define the virtual museum as a digital extension of the traditional museum and limit the resources of virtual museums to only include objects originally created in digital formats [6]. According to another definition, a virtual museum is “a virtual environment reproducing a space, which constitutes a reconstruction of a real place and/or acts as a knowledge metaphor, where visitors can communicate between them, and explore, create or modify spaces and (digital or digitalized) objects” [13]. This definition does not limit the functioning of the virtual museum to the World Wide Web (WWW) or any particular media technology, nor does it describe the objects presented in virtual museums as mere substitutes for analogue artefacts. Nonetheless, the effort to create a single universal definition is ongoing, and various authors are offering new proposals to deal with the topic of the virtual museum. In this article, the virtual museum refers to a virtual space created by a cultural institution, which collects digital reproductions of institution’s collections and to which the public has access using the Internet.

It is also true that although the concept of virtual museum has been discussed for some time, virtual museums were perceived in society only as a substitute for an ordinary, in-person cultural participation, and thus the development of the idea of virtual museum was slow. Only some cultural institutions – usually the most important and renowned, with substantial budgets – involved themselves in creating virtual exhibitions or digitised some of their collections. For example, in 2009 the Europeana web portal created by the European Union started its activities, providing access to hundreds of thousands of cultural heritage artefacts from institutions across Europe, including artworks; books; music; and videos on art, newspapers, archaeology, fashion, science and sport [14].

The idea of a virtual museum also has a clear political meaning. Linked with the premise of broad and free Internet access, virtual museums remove obstacles to accessing cultural heritage and indulging in art. Hence, a virtual museum is in its nature an egalitarian vision, addressing social exclusion. There are a number of possible reasons why individuals are reluctant or even unable to participate in culture [15] and, more specifically, visit museums. Sometimes it is lack of examples and habituation, resulting in a lack of interest, but it may also be personal financial situation or even a lack of public transportation. A virtual museum seems to promise to overcome all these obstacles by giving every person, wherever located, the opportunity to access cultural items and collections from their own smartphone or other personal electronic device. Thus, the idea of a virtual museum is inseparably linked to the broader concepts of cultural democracy, cultural pluralism and audience development, which include creating and promoting cultural opportunities for everyone. These concepts also refer to encouraging not only more people, but also a more diverse group of people to enjoy the cultural offer, for which, in turn, it is essential to know who the people who consume art are and also what they expect [16, 17]. As can be seen, the virtual museum is a powerful emancipatory tool that may be used for the creation of a more educated and egalitarian global society – an idea worth of pursuit in and of itself.

However, it is only thanks to the pandemic that perceptions of the role and importance of virtual museums have significantly changed. During national lockdowns, to encourage audiences to at least virtually visit their premises, museums undertook various steps to make their collections available online, including offering virtual walks and guided tours [18, 19]. Within a short period of time, museums had to redefine their objectives and operating principles as the pandemic “has caused in museum practice a backward step towards a previous practice” [20] by requiring museums to suddenly “re-become archives preserving objects and meanings” [20]. Fortunately, modern technology stepped in and helped museums to retain their role as more than mere archives, but in so doing cultural institutions faced a serious challenge in the form of copyright.

## Copyright in Museums

Before beginning discussion on the obstacles created by copyright, it is worth emphasising that copyright is not the only legal aspect that can significantly affect the activities of museums, especially virtual ones. In addition to copyright, contract law or data protection also plays a significant role, but this article is dedicated specifically to copyright (and related rights).^3^

### Copyright as a Branch of Law

Copyright is a branch of civil law, and, as part of intellectual property law, it is intended to protect the interests of authors and regulate the use of their works. Its existence is justified, on the one hand, by “natural rights-based ownership of the fruits of one’s intellectual labors” [21:1] and, on the other hand, “utilitarian expectations that granting authors property rights in their works will encourage them to create works for the greater benefit of society” [21:1]. The copyright doctrine distinguishes between two models of copyright law: monistic and dualistic. The dualistic model distinguishes between moral and economic rights, whereas the monistic model treats them as a single and non-transferable right of the author [22, 24–25]. Economic rights aim to guarantee their holder remuneration for others’ use of their work. The rights holder can therefore decide by whom and how the work may be used as well as its reproduction, public communication and distribution. In contrast, moral rights protect the bond between the creator and the work [23]. They consist of the right to be recognised as the author of a work, the right to the integrity of a work, and the right to decide when and how a work is first made available.^4^

The subject of a copyright is the *work*, which is an intangible property and is understood to be the result of the creative activity of a human individual [21:4]. The artistic or commercial value of the work is irrelevant, and even objects commonly regarded as kitsch are protected. For copyright protection to exist, it is necessary to express or fix the work in a tangible form, which means that “the work must be embodied in a copy which allows it to be seen or copied by others” [24:3], but no other formalities are required. For example, a fixation of a poem could be to write it on a piece of paper. Although copyright protection begins with the creation of the work, it does not last forever. According to the Berne Convention, which sets minimum copyright standards, protection must be granted until the expiration of the 50th year after the author’s death.^5^ In many jurisdictions, however, this period has been extended to 70 years [21:4]. Copyright is also not absolute. There are many exceptions as well as limitations. Some results of human activity are explicitly excluded from copyright protection by their sheer nature. For example, scientific discoveries or philosophical ideas are not a subject for copyright, not only because it might be seen as immoral to limit their use by others but also because it may be difficult to provide efficient legal protection. Likewise, moral reasoning and important social interests limit the rights of authors in cases of obligatory statutory limitations or what is termed fair use, which is when the work is reused or cited in a special context, for example, in journalism or for scientific purposes. In any case, however, for authors’ rights to be respected, restrictions must meet the ‘three-step test’, which is based on the wording of Article 9(2) of the Berne Convention.^6^ The first step states that limitations and exceptions must be restricted to ‘certain special cases’, meaning that they must not be excessively broad. Secondly, they cannot conflict with a normal exploitation of the work. In other words, they cannot deprive the rights holder of an actual or potential source of income. Thirdly, they cannot unreasonably prejudice the legitimate interests of the author, which means that they must not cause disproportionate harm to the rights holder, including materially [25].

### Copyright Restrictions on Museums’ Activities

In scholarly literature it is emphasised that copyright affects all spheres of museum activity [26:38]. Currently, the range of activities carried out by cultural institutions is wide and encompasses not only providing access to collections, organising exhibitions, and education in the broad sense of the term but also promotional and publishing activities, commercial services, and the increasingly frequent transfer of the traditionally understood exhibition activity to the Internet. From the legal perspective, whether cultural artefacts in an institution’s collection can be used in its activities such as exhibitions, digitisation or digital exploitation depends first and foremost on whether they are qualified as works that fall within the parameters of copyright. Thus, not every museum piece – understood as a movable or immovable thing owned by a museum, registered in the inventory of museums and potentially constituting a national asset – will be considered a work from the copyright perspective. The use of museum pieces not deemed ‘works’ under copyright is much simpler because the institution does not have to consider its copyright aspects.

However, if an artefact is protected, it is also necessary to distinguish the work itself from its physical carrier. The acquisition by a museum of ownership of an object constituting a work does not result in transferral of the economic rights to the work; this is due to the fact that from the legal perspective, a work is an intangible (immaterial) good. As a consequence, there must be a separate legal basis for using the work physically embodied in the object owned by a museum. This basis may be a licence or an agreement transferring copyrights, both being a separate agreement between an author (or their successor in title) and a museum. In principle, a contract transferring copyrights is of a permanent nature and transfers the economic rights to another person, who thus obtains all economic rights to the work. A licence agreement, on the other hand, only permits a specific use of the work (for a specific period of time and in a specific territory), while the rights remain with the author or another authorised person. National laws may differ on the mandatory form of these contracts or on the time period for which they may cover. There is nothing to prevent museums from concluding the relevant copyright agreements at the time of transferring ownership of the artefact to define the extent to which they can use the work; doing so can certainly mean fewer problems for them in the future. However, it is also possible to envisage situations in which this is not possible (e.g., the acquisition of the artefact from a person other than the current copyright holder); in these cases, an agreement will most likely have to be concluded afterwards, perhaps only when the need to use the work arises and with someone other than the previous owner of the tangible medium. Conversely, sometimes it is also possible to use the work freely, for example, under the provisions of exceptions and limitations in copyright law or even outside copyright law after the expiry of the economic rights to the work.

To further complicate matters in this already complex legal landscape, national jurisdictions may differ in exceptions and limitations in copyright law. For this reason, certain regulations were proposed at the EU level to allow museums and other cultural institutions to use works from their collections without obtaining the consent of the rights holder. The following sections detail the relevant legal acts.

### Exceptions to Copyright in European Law

#### Infosoc EU Directive

Directive 2001/29/EC of the European Parliament and of the Council of 22 May 2001 on the harmonisation of certain aspects of copyright and related rights in the information society (hereinafter: Infosoc Directive) introduces a number of mandatory as well as optional exceptions to copyright that Member States may or may not have implemented into their legal systems. Exceptions set out in Article 5 of the Infosoc Directive to the right of reproduction and the right of communication to the public of works are the most relevant to museum activities. Limitations include *inter alia* use of works such as works of architecture or sculpture made to be located permanently in public places, use for the purpose of advertising the public exhibition or sale of artistic works to the extent necessary to promote the event (excluding any other commercial use), and reproduction of works for the purposes of preservation. It is important to note, however, that the scope of the exceptions created in Article 5 was implemented in national copyright laws with substantial differences regarding, for example, format or the number of allowed copies [27, 25–28].

#### Orphan Works

So-called orphan works constitute the second type of permitted use regulated in EU law, this time by Directive 2012/28/EU of the European Parliament and of the Council of 25 October 2012 on certain permitted uses of orphan works (hereinafter: Orphan Works Directive). Orphan works are works for which copyrights have not yet expired, but it is impossible to reach the holders of these rights for various reasons – they may not be known, or it may not be possible to contact the rights holder or their heirs. If a museum has such works in its collection, using them (e.g., by making them available or reproducing them) might expose it to an infringement of copyright. Consequently, because of museums’ fear of possible consequences of copyright infringement, orphan work status can present a substantial obstacle to preserving cultural heritage [28:286–304].

The reason why orphan works occur is because copyright protection is relatively long and economic rights are transferable. As such, it may be difficult to identify the person holding a relevant right to a piece in a museum’s collection. The author may transfer their rights to a third party or heirs, or the so-called ‘fragmentation’ of rights may happen due to turbulent events. In Poland, for example, from 1945 to 1990 many publications were created underground as a part of a so-called second-circuit of culture in which authors remained anonymous as they opposed the oppressive government. It is reasonable to believe that the scale of the phenomenon of orphan works is large. For example, the German National Library estimates that orphan works in the library’s collection include approximately 585,000 books, 138,000 phonograms and 50,000 film works [29:25]. The British Library estimates that orphan works account for 40% of copyrighted works in its entire collection [30:256–257]. On the European scale, about 13% of protected book publications are considered orphan works [31:1354].

The Orphan Works Directive does not cover all categories of works. Article 1 restricts it to works published in the form of books, journals, newspapers, magazines or other writings; cinematographic or audio-visual works; and phonograms. Importantly, these works must be in the collections of publicly accessible libraries, educational establishments, museums, archives, institutions responsible for film or audio heritage, or public-service broadcasting organisations as only these institutions have been authorised to use orphan works in the public interest. Thus, both the scope of works that can be granted orphan status and the entities entitled to use them is rather restricted.

An appropriate search (called ‘diligent’ in the Orphan Works Directive) for the rights holder is an important part of the legal framework regulating the use of orphan works. Only a lack of success in finding rights holders despite a diligent search makes it possible for a work to be granted orphan status. This issue is a subject of Article 3(1), which states that the search should be made for each work in good faith and should take place prior to the use of the work in question. It should be carried out by a legitimate institution listed in the Orphan Works Directive (publicly accessible libraries, educational establishments and museums, archives, film or audio heritage institutions and public-service broadcasting organisations), but entrusting this task to a third party is not forbidden. The Orphan Works Directive also foresees in Article 3 that a search shall be in principle carried out in the Member State where the work was first published or broadcasted. Moreover, a comprehensive list of sources that supply information on the works is included in the Annex to the Orphan Works Directive, and these need to be consulted for each category of works. The Orphan Works Directive leaves a certain degree of freedom in this regard because the appropriate sources are determined by each Member State, in consultation with rights holders and users, and include at least the relevant sources listed in the Annex. As an example of the extent of the list of these sources, sources for published books according to the Annex include legal deposits, library catalogues and authority files maintained by libraries and other institutions, publishers’ and authors’ associations in the respective country, existing databases and registries, WATCH (Writers, Artists and their Copyright Holders), ISBN (International Standard Book Number) and databases listing books in print, the databases of the relevant collecting societies and particularly reproduction rights organisations, and sources that integrate multiple databases and registries such as VIAF (Virtual International Authority Files) and ARROW (Accessible Registries of Rights Information and Orphan Works).

Similarly extensive lists have been defined for other categories of works as well. Therefore, performing a diligent search is a lengthy and complicated process, especially in situations where there are several rights holders and all have to be approached. Additionally, orphan status is not permanent. According to Article 5, Member States must ensure that a rights holder of a work or phonogram considered to be an orphan work has, at any time, the possibility of putting an end to the orphan work status; this is a huge inconvenience resulting in legal uncertainty. A cultural institution may put a lot of time and financial effort into a diligent search for a rights holder only to find later that the established orphan status has been lost and that institution is obliged to pay compensation for an unauthorised use of the work. Per Recital 18 of the Orphan Works Directive, the amount of compensation depends on the nature and extent of the use, the amount of revenue generated and the harm caused to the rights holder by that use. Although the change in the work’s status does not in every case mean that the institution cannot continue to use the work by obtaining consent from the disclosed rights holder [32], the uncertainty caused by the regulations regarding orphan works is substantial.

The permitted use of orphan works is also very limited as the use is available to the narrow circle of entities and works that are already in an institution’s collections, as well as permitted only for the performance of their statutory tasks, and limited to reproducing and making available in such a way that the public may access the works from an individually chosen place and time [33:144]. Furthermore, it is not possible to use orphan works for commercial purposes. An institution may only earn revenue from the use of orphan works as long as it is used to cover the direct costs of digitising and making them available to the public.

The limitations indicated above lead to a negative assessment of the Orphan Works Directive in the context of this paper. The diligent search process is simply too time-consuming and costly for museums, which often lack the necessary human resources for conducting the diligent search due to staff shortages. The lack of clear criteria for diligence in conducting searches means that cultural institutions prefer not to risk a possible infringement of the law. The excessive restriction of the entities that could benefit from the granting of orphan status to a work, and the fact that the category of works to which the provisions can be applied is too limited, has the result that the Orphan Works Directive is not used as much as it could be and probably much less than expected. Despite this critical assessment, one cannot forget that the Orphan Works Directive was the first legal act to deal with the issue and a very important step towards comprehensive regulation of the use of orphan works.

Among the proposals for change were those for extending the circle of authorised entities to private individuals and even entrepreneurs, abolishing the liability of institutions when a rights holder comes forward to eliminate concerns about legal consequences and allow commercial exploitation [34]. In Article 10, the Orphan Works Directive contains an extensive review clause that suggests directions for further changes.^7^ In fact, in 2020 a survey was carried out among EU Member States on the application of the Orphan Works Directive [35]. Since this was already after the adoption of the DSM Directive, particular attention was given to overlapping regulations. As discussed later in this article, many proposals made regarding the Orphan Works Directive have lost their relevance with the entry into force of the DSM Directive, and some scholars even doubt that the former directive should remain in circulation.

### Digitisation and its Limits

Although the digitisation of collections had become increasingly important for museums even before the pandemic, during the coronavirus outbreak it became crucial. The main reason for the digitisation of collections is, as mentioned, to allow broad access to cultural heritage in the form of virtual museums. However, while the idea of the virtual museum worked well in the time of the pandemic, copyright remains an important factor that cultural institutions must take into account while digitising their collections to share them online. “The prerequisite for the ‘virtual museum’ are digitized data” [36:191], and digitised data must fulfil certain legal requirements to be shared.

Hence, the virtual museum is an area where public interests, such as the need to safeguard cultural heritage and the desire to make it accessible to the widest possible audience, and the interests of private stakeholders, protected by copyright, are clearly in conflict. The article now characterises some of the obstacles that cultural institutions must overcome in order to digitise their collections on the way to creating virtual galleries or museums.

The first problem consists of the fact that even the term *digitisation* has not yet been defined across the EU legal system. A noteworthy definition was proposed by the Court of Justice of the European Union (hereinafter: CJEU) in C-117/13, which stated that “the digitisation of a work, which essentially involves the conversion of the work from an analogue format into a digital one, constitutes an act of reproduction of the work” [37]. It is assumed that this is “the creation of a digital record of the content of various types of documents (materials) existing in more traditional, non-digital forms, such as, in particular, printed documents, and by means of appropriate techniques, such as for example scanning” [38:19]. In the absence of separate regulations applying only to digitisation, one should conclude that digitisation will fall within the scope of exploitation of the work, and hence all rules pertaining to exploitation will be applicable to digitisation as well.

Hence, it is necessary for a museum to decide whether it has the right to digitise a particular object because “[d]igitizing copyrighted works and making them available online involves the rights of reproduction and of communication to the public, each of which usually requires permission from the copyright holder” [27]. Only in the case of works belonging to the *public domain* are there no restrictions on digitisation. The term *public domain* is used to describe works for which no intellectual property rights exist. The public domain includes manifestations of creativity which do not have an individual character, meaning those which have never been protected, such as names or short slogans, as well as objects excluded from protection by legislators, such as texts of normative acts. Finally, the public domain includes works for which copyright protection has expired because the required time period has passed.

In all other cases outside the scope of public domain, a museum has to obtain authorisation from the author, for example, by signing an appropriate contract, or digitisation may take place on the basis of legal provisions introducing copyright exceptions where the author’s permission is not needed. However, as seen from the examples above, there is no exception in EU law that explicitly allows museums (or cultural institutions more broadly) to freely reproduce works from their collections.

### Copyright Status of Digital Reproductions

Once the question of a work’s copyright status is resolved and a reproduction of it for the purpose of a virtual museum has been legally created, the reproduction itself can sometimes be protected as well. Although in principle the idea of museum photography is to reproduce the original as faithfully as possible, which leaves little room for creativity and individuality to the author of the photograph [39] (and thus a faithful museum photograph with only documentary purposes will not qualify as a work), it may happen that the photographer had enough freedom in the choice of lighting, angle or composition when taking the picture that their photograph is protected as a derivative work and so any further use of it will require the rights holder’s consent.^8^ A good example of this is the photography of 3D objects such as sculptures. Their peculiarity means that, in some cases, it is possible to question the passive role of the photographer. Prof. Yaniv Benhamou asks even more far-reaching questions in this regard:

“This all seems straightforward, but is it? Do new high-resolution digital cameras, which enable users to adjust pixelation, light and contrast, allow them to express the individuality and originality of their work?” [27].

Moreover, there are also differences between jurisdictions. Some jurisdictions have introduced provisions protecting even non-creative photography via the legal institution of related rights. EU countries where non-original photography is protected include Germany, Italy and Spain [40].

As demonstrated, copyright provisions create serious obstacles to realising the vision of a virtual museum. Thus, it is presumed that this conflict between copyright protection and the mission of cultural institutions is the reason for the regulation of certain copyright aspects in the DSM Directive.^9^

## DSM Directive

Although the DSM Directive was devised as a necessary step towards the harmonisation of copyright law in the European Union, its content was disputable from the very beginning. Especially due to the content of Article 17, opponents quickly called the Directive ‘ACTA 2’^10^ and claimed that it would lead to the censorship of the Internet.^11^ On the other hand, for certain groups the passing of the DSM Directive was perceived as a great success .^12^ Member States are obliged to bring into force the laws, regulations and administrative provisions necessary to comply with the Directive by 7 June 2021^13^.

Despite the suggestive title, the Directive does not comprehensively regulate copyright in the Digital Single Market but rather touches on various issues of importance [44:21, 45:5]. Its provisions are relevant to text and data mining for scientific research, the use of works in digital and cross-border teaching activities, the activities of cultural heritage institutions for the preservation of works in their collections, the use of out-of-commerce works, collective licensing with extended effect, the protection of press publications, equitable remuneration of authors, and the protection of material resulting from reproductions of works of visual art in the public domain. A number of provisions included in the DSM Directive directly affect the functioning of museums and other institutions from the cultural sector. Some explicitly identify those institutions as beneficiaries of the limitations and exceptions to copyright law that the DSM Directive introduced (e.g., Article 6 on the preservation of cultural heritage, Articles 8–10 on the use of out-of-commerce works), while others introduce general rules that museums can rely on in their everyday practice (e.g., Article 5 on the use of works in digital and cross-border teaching activities, Article 12 on collective licensing with extended effect). Article 14 relating to works of visual art in the public domain responds to the problems of appropriation of the public domain that have arisen in connection with the activities of museums and art galleries and that are discussed in the following paragraphs.

The Europeana Foundation, which supports the cultural sector in adapting to digital change and works for universal access to heritage resources, notes that “[t]he Directive recognises the cultural heritage sector’s important role in the digital world. It makes it easier to digitise and share cultural heritage for the benefit of research, education, literacy, creation and culture” [46]. The Foundation praises the place that the exceptions to copyright relating to cultural heritage institutions are given in the DSM Directive and remarks that “European legislators have made an effort to adapt these to the digital world, and to harmonise them across the European Union to ensure they work across-borders” [46]. Hence, it seems reasonable to claim that the DSM Directive provides – at least to a certain extent – a legal framework that is useful for regulating the activities of cultural heritage institutions in the Digital Age. Despite the positive reception, however, specific doubts on the way to the creation of virtual museums have still emerged.

### Definition of Cultural Heritage Institutions

Cultural heritage institutions are defined in Article 2 of the DSM Directive, thus becoming an autonomous concept of the EU law. ‘Cultural heritage institution’ means a publicly accessible library or museum, an archive, or a film or audio heritage institution. Thus, museums are explicitly mentioned in this definition. In Recital 13, on the other hand, the concept of cultural heritage institutions is clarified to cover publicly accessible libraries and museums regardless of the type of works or other subject matter that they hold in their permanent collections, in addition to archives and film or audio heritage institutions. The definition does not specify the legal form or structure of an institution to be considered a cultural heritage institution. Consequently, this matter is determined by EU Member States’ national regulations [47:2]. Leaving the task of defining the key concept of cultural heritage institution to the Member States may lead to discrepancies and practical problems in the application of the Directive provisions. Entities may qualify as cultural heritage institutions in one national jurisdiction but not in another. To remedy this, the DSM Directive was supposed to stabilise the legal status of museums and other institutions from the cultural sector across the EU, but the vague definition in its provisions may instead cause confusion.

### Preservation of Cultural Heritage

The first provision of the DSM Directive directly addressing cultural heritage institutions is Article 6. The reasons for this provision are explained in Recitals 25–30 of the preamble, where the importance of preserving collections for future generations and the necessity of copyright exceptions are underscored. Article 6 states that the Member States shall provide for an exception to specifically defined rights^14^ in order to allow cultural heritage institutions to make copies of any works that are permanently in their collections, in any format or medium, for purposes of preservation. Making copies for the purpose of preserving cultural heritage is an example of the permitted use of any work from a museum’s collection [44]. The implementation of this exception is mandatory, which constitutes a new approach in EU copyright legislation. Up to this point, Member States had discretional power to choose which exceptions to exclusive rights they would implement in their legal systems [48]. Article 6 ensures that all cultural heritage institutions across the EU can make copies of works from their collections for preservation and protection purposes [49].

As such, the rights of cultural institutions are extended from previously existing regulations, which only allowed copies of works to be made to protect them from destruction and often did not provide for digitisation or did not apply to works that were in digital form from the start [50]. However, while Article 6 allows for reproduction, it does not allow sharing of the reproduced works. Without sharing, the idea of a virtual museum is pointless.

Article 6 should be interpreted broadly and should not limit cultural heritage institutions in time, format or the extent to which the preservation of works is guaranteed [48]. Specifically, cultural heritage institutions are not obliged to wait until there is an imminent risk of the disappearance or destruction of the work in question. This is a vast change as many national laws prior to this have only referred to reproduction for the purposes of reconstruction or replacement of a lost work. Institutions are also free to choose the tools, means and technologies of reproduction [51]. Article 6 does not limit copying to digital copies only – analogue and even three-dimensional copies are also possible [44]. In contrast, the Infosoc Directive discussed above provides for the possibility of making copies of works collected by museums but only to a very limited extent. In addition, Member States were not obliged to introduce its provisions, and, consequently, there was no full harmonisation of the law in the area of permitted use relevant for museum practice.^15^ Article 5(2)(c) of the Infosoc Directive established the possibility of a limitation or exception to the reproduction right for specific acts of reproduction not for direct or indirect economic or commercial advantage made by publicly accessible libraries, educational establishments, museums or archives. Nevertheless, even in those countries that have implemented the relevant exception in national law, additional limits have oftentimes been introduced regarding formats or numbers of copies made, thus creating obstacles for museums’ practice [51].

In the case of the DSM Directive, Article 6 is limited to works or other protected subject matter contained permanently in the collections of cultural heritage institutions.^16^ Recital 29 explains that this is the case when copies of works or other subject matter are owned or permanently held by an institution, for example, as a result of a transfer of ownership or a licence agreement, legal deposit obligations or permanent custody arrangements. This catalogue seems to be only an exemplary one and other forms of permanent possession may be added, rendering the content of Article 6 quite broad and entitling museums to proceed with digitisation. Yet many cultural heritage institutions entitled to make reproductions of works and other subject matter will not be able to make such copies due to lack of adequate technical or financial means. The EU legislator seems to be aware of this problem. Recital 28 notes that cultural heritage institutions should be allowed to rely on third parties acting on their behalf and under their responsibility for the making of copies. However, it is emphasised in the literature that to ensure the rights of rights holders in such situations, copies made by third parties have to be directly returned to the mandating institution, and any provisional or incidental copies must be immediately destroyed [52].

In turn, Article 7 of the Directive provides a strong guarantee for Article 6’s practical implementation; according to its ruling, any contractual provision contrary to the exception provided for in Article 6 shall be unenforceable. This unenforceability applies to any contract that might limit the applicability of the copyright exception provided by Article 6, letting cultural heritage institutions based in the EU ignore provisions in conflict with the exception [53].

#### Obstacles Remain

Based on the provisions characterised above, the situation of cultural heritage institutions in their efforts to digitise collections appears advantageous. Nonetheless, obstacles may still be identified on the way to a realisation of the idea of a virtual museum or gallery. For instance, technical protection measures normally used to prevent access to and use of protected works contrary to the will of the rights holder are often viewed as necessary to ensuring the protection and effective exercise of the rights of creators and other rights holders. Such measures use various types of technology to control who may access a digital work protected by copyright (e.g., password control, payment systems, encryption, captcha technology). However, they may also prevent the practical application of Article 6 because cultural heritage institutions that obtain the right to make a copy of certain works on the grounds of Article 6 are not automatically entitled to remove the technical protection measures implemented by the author or other copyright holders. In other words, the DSM Directive does not allow museums to remove technical protection measures from the works in their collection [53]. It is thus up to the rights holders to take action on a voluntary basis through the choice of appropriate measures to ensure that cultural institutions can benefit from Article 6’s exception. Only in the absence of voluntary action by the rights holder can cultural institutions turn to state authorities as the DSM Directive in Recital 7 empowers Member States to take appropriate measures to ensure the application of Article 6 in cases of protected content. Yet concerns have been raised that this mechanism may lead to significant delays because institutions must appeal to the rights holders to change or adapt each time they find themselves confronted with protection measures. In addition, the negative deterrent effect caused by technical protection measures may make institutions reluctant to start the whole procedure, which may complicate the application of Article 6 in practice [53].

Overall, due to its very limited scope of application and the fact that it does not resolve all dilemmas related to making works available in the digital environment, Article 6 should be assessed with caution. At the same time, its mandatory nature and the unenforceability of contracts limiting its application highlight the new approach to copyright exceptions in EU law, which have been reoriented to better safeguard public interests. For this reason, some authors consider Article 6 as the least controversial element of the DSM Directive [54:15].

### Out-of-commerce Works

The Orphan Works Directive is complemented by the provisions of the DSM Directive regarding so-called out-of-commerce works.^17^ The aim of these provisions is to enable the creation of digital collections of out-of-commerce works and their non-commercial sharing by the institutions that have them in their collections. A significant portion of collections of various cultural heritage institutions consists of works that have never been commercially available, and it is therefore difficult or even impossible for various reasons to obtain rights to copy or share them. As Recital 30 of the DSM Directive states, “[t]his can be due, for example, to the age of the works or other subject matter, their limited commercial value or the fact that they were never intended for commercial use or that they have never been exploited commercially”. Although these works do not usually have a commercial value, their cultural significance is often important [55:7]. For this reason, another mandatory exception to copyright was introduced by Articles 8 to 11 of the DSM Directive allowing cultural heritage institutions to make such works available without the permission of rights holders.

The DSM Directive does not impose any limitation on the types of works that may be considered out-of-commerce, and so these can include works in analogue form as well as those that were originally created in digital form [56:694]. The only requirement – identical to Article 6’s exception – is that cultural heritage institutions must have works permanently in their collections. The DSM Directive lists in Recital 37 examples of works that may have this status: “posters, leaflets, trench journals or amateur audiovisual works, but also unpublished works or other subject matter”. Out-of-commerce works should not be confused with either orphan works or works in the public domain. In order to determine whether a work is in fact out-of-commerce, it is necessary to make a reasonable effort to determine whether it is available to the public. The EU legislator has left it to Member States to determine who should make these efforts. However, it seems that in the case of the licensing mechanism of Art. 8(1), this responsibility rests with collective management organisations, while in the case of the exception from Art. 8(2) and (3), it rests with the cultural heritage institution interested in using a given work [44:105]. Verification of the accessibility of a work should be a single process and should be carried out in the Member State in which the cultural heritage institution is located.

Hence, Article 8 of the DSM Directive provides for two solutions: a principal solution and a ‘fallback’ solution. The first solution implies that collective management organisations may conclude a non-exclusive licence for non-commercial purposes with a cultural heritage institution for the reproduction, distribution, communication to the public or making available to the public of out-of-commerce works that are permanently in the institution’s collection, irrespective of whether all rights holders covered by the licence have mandated the collective management organisation. The assumptions here are that the collective management organisation is sufficiently representative and all rights holders are guaranteed equal treatment in relation to the terms of the licence; this means that it is prohibited to vary the conditions under which a licence to use a particular work in the same fields of exploitation will be granted, such as “with regard to the territorial scope of the licence, the licence fees and the permitted uses” [44:97]. The problem is that the DSM Directive does not specify what characteristics a collective management organisation should meet in order to be considered sufficiently representative; instead, it is left to the Member States to determine the representativeness criteria.^18^ This is crucial because determining whether and which organisation meets the representativeness requirement is key to deciding which mechanism – licence from Art. 8(1) or exception from Art. 8(2) and (3) – an institution is eligible to use.

In some cases, the scope of application of this licensing mechanism overlaps with Article 6 of the Directive. In such cases, no separate licence are required for the reproduction of out-of-commerce works for the purposes of the preservation of cultural heritage – only a licence for making works available to the public may be required [57]. In contrast, the second solution introduced by the provisions of Article 8(2) and (3) regards cases where there is no collective management organisation that could grant an appropriate licence to the cultural heritage institution in accordance with Article 8(1). The scope of this exception is narrower as it covers only making works available to the public instead of reproducing, distributing, communicating to the public or else making works available to the public as foreseen in Article 8(1) [44:98]. Additional requirements to benefit from this exception are indicating the name of the author or any other identifiable rights holder (unless this is impossible) and that works or other subject matter are made available on non-commercial websites [44:98].

In summary, a cultural heritage institution intending to use out-of-commerce works first has to establish whether the work is permanently in its collection. The next step is to contact the relevant collective management organisation which can grant a licence authorising the use of the works. If a licence is not granted, it is not possible to exploit the works. If a competent organisation does not exist, a cultural heritage institution may consider employing Article 8(2). In both cases, it is necessary to guarantee the rights of the rights holders. It is necessary to publish information on the works used and to provide availability of an opt-out mechanism.^19^ The whole procedure, compared to that set out in the Orphan Works Directive, is less complicated and does not require as many resources or as much work or time. Unlike orphan works, mechanism described above thus creates the opportunity for out-of-commerce works to actually be employed by cultural heritage institutions.

Still, major criticism has been expressed regarding the DSM Directive’s regulation of out-of-commerce works. Among the most significant drawbacks of the regulation, it is pointed out that cultural heritage institutions may find it difficult to identify the out-of-commerce works they have in their collections because their definition in EU legislature is not precise. In addition, the collective management organisation approached by the cultural heritage institution may not be interested in granting a licence, resulting in the impossibility of using the works in question as the DSM Directive does not provide for compulsory licences in such cases. There are also concerns that even when collective management organisations may be willing to grant a licence, the proposed fees will prevent cultural heritage institutions from signing an agreement. There is also a risk that after cultural heritage institutions have invested time and money in digitising and making works available, exclusive rights holders will express a desire to opt out [49]. While it is emphasised that this is unlikely to be a mass phenomenon, institutions have to bear all of these risks in mind [58:139].

Minimising these disadvantages depends mainly on how the provisions of the Directive are implemented by Member States. In accordance with Article 11, Member States shall consult rights holders, collective management organisations and cultural heritage institutions before establishing specific requirements pursuant to Article 8(5). In the literature, it is believed that the usefulness of the Directive will depend on the particular provisions prepared at the Member-State level, which are in turn dependent on the quality of the collaboration between cultural heritage institutions and collective management organisations [49]. That usefulness will be the key factor in determining the extent to which the DSM Directive will be preferred to the legal mechanism provided by the Orphan Works Directive since a particular work may qualify as both an orphan work and as an out-of-commerce work. Hence, a cultural heritage institution may choose which mechanism to use. If priority is given to stability, a diligent search for the orphan status of a work may be favoured despite its time-consuming nature [54:15, 59:791]. Only time will tell which provisions are more useful.

### Works of Visual art in the Public Domain

Interestingly, although copyrights protection is usually recognised as an obstacle to creating digital collections, sometimes it is used by museums to block others from reproducing digitised works from their collections. Museums and other cultural institutions often require third parties to obtain their permission to make or distribute reproductions of objects in their collections, including those that are not subject to copyright protection. This practice may be explained by the fact that digitisation of museum’s collections is costly, so the reservation of rights to a digital reproduction is supposed to be a compensation for the incurred costs [60]. It is ironic that museums – which suffer the most from copyrights – are the very institutions that try to stretch copyright protection even to photographs capturing works of art from their collections which are sometimes not protected themselves by copyright. It seems to be in conflict with not only the public’s expectation of unrestricted access and free use of cultural goods but also with the principles of copyright protection.

A prominent example of a museum restricting access to digital reproductions of its collections was the 2009 dispute between the National Portrait Gallery (NPG) in London and the Wikimedia Foundation [61]. The NPG digitised parts of its collections and made them available on the museum’s official website. In 2009, Derrick Coetzee downloaded more than 3,000 high-resolution images from that website and uploaded them to the Wikimedia Commons collection [62:1]. According to British law, the photographed images had been in the public domain for a long time since the time required for the expiry of copyright had already elapsed since their creators’ deaths [63]. However, the NPG sent a request to Derrick Coetzee to remove the uploaded images, invoking its copyright over the reproductions, due to the expenses incurred in the digitisation process. The dispute ended amicably, and the NPG chose not to pursue its rights in court [62:2]. The reason for this may have been the 1998 US court ruling in Bridgeman Art Library v. Corel Corp [64], in which the court stated that a picture that is merely a copy of another work is not original and therefore does not meet the prerequisites for copyright protection, and no new rights arise for reproductions of public domain works [62:2]. The NPG and the Wikimedia Foundation started a dialogue on the possibilities of using digital resources to find a mutually satisfactory solution. The result was the NPG granting tens of thousands of free Creative Commons licences and making tens of thousands more images available for educational purposes, for which the NPG only requested donations.

In 2015, the UK Intellectual Property Office issued a document entitled “Copyright Notice: Digital Images, Photographs and the Internet”, which, in reference to CJEU case law, stated that only original expressions of human activity that are the creator’s own intellectual creation are protected by copyright, and therefore digital reproductions of works with the purpose of merely faithfully reproducing the original cannot be protected [65].

Another well-known example of the practice of restricting access to reproductions of public domain works, which was one of the reasons why this issue was addressed in DSM Directive, is the ruling of the German Federal Court of Justice on 20 December 2018 in the case Museumsfotos [66]. In its judgment, the Federal Court of Justice had to settle a dispute between the Reiss-Engelhorn-Museum in Mannheim and Andreas Praefcke, a user and editor of the Wikipedia website, regarding his scanning and uploading of 37 photographs of paintings from the museum’s collection to Wikimedia Commons. 17 of the photographs were taken in 1992 by an employee of the museum and placed in the museum catalogue, and the other 20 were taken by the defendant during his visit to the museum in 2007. All the photos were scanned by the defendant, placed in a publicly accessible photo database and marked as being in the public domain. The Federal Court of Justice ruled that although the images photographed were already in the public domain, the photographs taken by the museum employee were protected because the person taking them had the opportunity to decide, among other things, on perspective or lighting. In relation to the photographs taken by the defendant, on the other hand, it stressed that there had been a breach of the ban on photography in force on the museum’s premises, and thus there was a breach of the contract between the museum and visitors in the general terms and conditions of visiting the institution; as a result, the museum could claim damages and stop online publication of the unlawfully taken photographs [67]. The defendant invoked fundamental rights, such as the right to information, which would allow him to take photographs for his private purposes. However, the Federal Court of Justice did not see any infringement in this regard. Instead, it ruled that photographs of paintings in the public domain and other two-dimensional works of art do not qualify for recognition as works but are protected by related rights. In fact, German legislation on the protection of photographs applies the so-called mixed system in which ordinary, non-original photographs may be protected but are only subject to a related right [68], regulated in § 72 of the German Copyright and Related Rights Act [69]. According to this provision, so-called other photographs (Lichtbilder) that do not have the characteristics of a work are protected by a related right, which in principle expires 50 years after publication. Article 6 of Directive 2006/116/EC allowed Member States to introduce provisions protecting photographs other than those that are an author’s original intellectual creation.

On the one hand, the cited ruling of the Federal Court of Justice confirms that the photographs at issue lacked the element of originality and were subject to weaker protection. This conclusion deserves a positive assessment as it is in line with the criteria developed by the CJEU that describe which conditions must be met to recognise a manifestation of human activity as a work. On the other hand, the ruling shows that the very existence of provisions allowing for the protection of non-original photographs may lead to legal uncertainty and cause problems in cross-border and online use as the same reproduction could be protected in one Member State and be in the public domain in another [70].

Taking both cases together, there was a need for uniform regulation at the EU level to ensure that all users are able to benefit from reproductions of works in the public domain without restriction and to prevent further appropriation of the public domain by cultural institutions. The EU legislator’s response can be found in Article 14 of the DSM Directive. According to this provision, reproduction of works of visual art which are in the public domain cannot be subject to copyright or related rights unless they are a new and original intellectual creation. As stated in Recital 53 of the Directive, “the circulation of faithful reproductions of works in the public domain contributes to the access to and promotion of culture, and the access to cultural heritage”. Thus, the copyright protection of such reproductions is not only “[…] inconsistent with the expiry of the copyright protection of works”, but inconsistent with the public mission of cultural heritage institutions, too. The intention of the EU legislator, who was relatively late in introducing Article 14 to the DSM Directive,^20^ was firstly to emphasise that once the economic rights to a work have expired, it can be reproduced and used without the author’s consent. The second, more important intention was to ensure that no exclusive rights would be granted over a non-original reproduction of a work of visual art already in the public domain [71], and therefore cultural heritage institutions would not have any legal basis to claim copyright protection over it. The latter has already been considered in EU law as the criteria for granting copyright protection to expressions of human activity have been addressed by the CJEU on several occasions. In fact, CJEU has ruled that

“in order for subject matter to be regarded as a ‘work’, two conditions must be satisfied cumulatively. First, the subject matter must be original in the sense that it is its author’s own intellectual creation. In order for an intellectual creation to be regarded as an author’s own it must reflect the author’s personality, which is the case if the author was able to express his creative abilities in the production of the work by making free and creative choices” [72].

In the case of photography, the following remarks are made:

“the photographer can choose the background, the subject’s pose and the lighting. […] the framing, the angle of view and the atmosphere created. Finally, when selecting the snapshot, the photographer may choose from a variety of developing techniques the one he wishes to adopt or, where appropriate, use computer software. By making those various choices, the author of a portrait photograph can stamp the work created with his ‘personal touch’” [73].

With this understanding of the concept of a photographic work, a faithful reproduction will never be subject to copyright protection. The situation is different in countries that protect manifestations of human activity by related rights in addition to copyright law. Reproductions that are not characterised by originality and do not meet the criteria for copyright protection obtain protection on the basis of related rights, even if they are only a faithful copy of the work.

It is important to note that Article 14 does not introduce sanctions or mechanisms of effective enforcement for abusive use of exclusive rights to public domain works [40]. In essence, Article 14 prohibits protection by exclusive rights of uncreative (i.e., *faithful reproduction*) photographs and other forms of reproductions of works of visual art with expired copyright protection [44:131]. However, this does not mean an absolute prohibition on the protection of such photographs under related rights. Indeed, the DSM Directive has limited the scope of application of Article 14 to only include the ‘works of visual arts in the public domain’. It will not apply to reproductions of other creations that do not fulfil the characteristics of a work, and thus such reproductions can still be protected by related rights [44:134].

#### Ambiguities in Article 14

The DSM Directive does not define the concept of works of visual art in any way. This category of works usually includes paintings, drawings, photographs, sculptures, architectural works, crafts, wall paintings, graffiti or audio-visual works [70]. Yet it is not difficult to find borderline examples that may or may not fall within the scope of this provision. Such examples include maps, musical notations and technical drawings [44:133–134]. To complicate the matter further, the concept of works of visual art may be understood differently in particular Member States, thus making harmonisation of copyrights in the EU difficult [60]. The lack of an unambiguous definition will likely result in the CJEU being entrusted with the task of defining this category of works and resolving doubts that arise over the application of the Article.

Clarification is also required as to whether Article 14 will apply to reproductions of works of visual arts made when the original work is already in the public domain as well as those made while the original work is still subject to copyright protection. The wording of the provision does not determine this, so *lege non distinguente* it seems reasonable to apply it to both categories of material resulting from reproduction. In Member States protecting non-original reproductions by related rights, this will be relevant in determining whether such a reproduction will be protected at all at any point in time or whether it will enter the public domain from the very beginning. In the literature it is pointed out that in the case of reproductions made after the original work has entered the public domain, the reproduction can never be protected by related rights. Conversely, when the reproduction is made while the copyright protection of the original work is still in force, the protection of related rights can be granted, but only for the time remaining before the original work enters the public domain and in no case for longer than the duration of the copyright protection of the original work [70].

Some doubts may also arise as to how the concept of materials resulting from the reproduction of a work that do not constitute the author’s own intellectual creation should be understood. Article 14 applies to faithful reproductions of two-dimensional works such as photographs. However, it seems justifiable to also apply Article 14 to reproductions of three-dimensional works made in a way that preserves the exact form (e.g., casting, 3D printing), with no creative transformation of any kind introduced in the process [71]. The case is more complicated in the case of two-dimensional reproductions of three-dimensional works, which may often constitute the intellectual creation of the reproducer due to choices they make regarding, for example, the shot, angle or lighting with which a reproduction is created. Therefore, *ad casum*, it is necessary to establish the existence of elements of originality in such reproductions. Hence, the requirement of faithful reproduction formulated in Article 14 excludes such reproductions in which other elements in addition to the work of visual art are depicted [74].

Another set of doubts relate to the cases when reproductions cover only a part of a work of art. It is emphasised that Article 14 should apply to these, too, because otherwise it would be too easy to circumvent the regulation by deliberately cutting out even a small part of the original work [74]. The DSM Directive also makes no distinction between digital and analogue reproductions. Although it is the digital market that has become the main focus of the new EU copyright and related rights regulations, and it can be argued that Article 14 will only apply to digital reproductions, the lack of a clear distinction between these two categories should result in the application of the regulation in question to both digital and analogue reproductions. Furthermore, due to the significant variations between Member States’ legal systems regulating the protection of non-original reproductions through related rights, Article 14 has only been implemented in some countries.^21^ Member States in which related rights protection does not extend to non-original material resulting from reproductions of a work did not have to take any action in this regard.

While the copyright issues relating to faithful reproductions of works of art appear to be resolved by Article 14 and it seems reasonable to argue that museums can no longer prohibit making and sharing of reproductions of works from the public domain by merely invoking copyright, this does not imply complete freedom in photographing collections of works of art in the public domain or their digital reproductions. The DSM Directive does not address these issues in any way. There are many other reasons why cultural institutions may choose to prohibit the taking of photographs, even of collections that are already in the public domain. This may be justified on conservation grounds, reasons relating to visitor safety or even provisions arising from exhibit loan agreements. The possibility of deciding whether and to what extent the photographing of collections is allowed may also be derived from the right of ownership and the possibility of regulating the scope of use of an exhibit in an administrative act, such as the museum’s regulations. In the Museumfotos case, the Federal Court of Justice emphasised that the museum had a legitimate interest in regulating the behaviour of visitors, and this includes the possibility of imposing a ban on photography to “protect artworks and visitors’ privacy, to facilitate the proper running of the museum, and to honour other general obligations to lenders and the public” [60].

Ultimately, it is worth emphasising that although the DSM Directive is usually assessed with reservations by scholars, Article 14 has been acclaimed as a provision that in fact protects the public domain from erosion [70]. Article 14 is the first regulation in the *acquis communautaire* where the term *public domain* is explicitly mentioned [40], which may herald the creation of a legal framework that can effectively protect the public domain.

## Conclusion

With the pandemic and the rising importance of online services, including in the public and cultural sectors, comes the hope for the practical realisation of virtual museums and galleries. However, closer analysis of developments in EU law and copyright reveals that virtual museums are still a long way from replacing traditional museums. There are legal, financial and practical obstacles to the process of digitising collections. Some obstacles are related to the everlasting conflict between free access to works of art demanded by the public and moral and economic interests behind the process of creation, collection and exhibition. EU law is well behind practice, and the harmonisation of national regulations is not yet reality.

The urgent task for legislators in the near future will be to create a legal framework that gives museums more freedom in the process of digitisation and making their collections available online. It will likely require years to adapt the legal system to the new reality dominated by the Internet and digital phenomena, but that process is necessary. As indicated in this article, some steps in this direction have already been taken, but this goal may never be reached. After all, interacting with art in person is a completely unique experience that cannot be easily replaced by looking at pictures on a computer screen; even the best digital reproduction will never replace an original work of art.^22^ Nor can the social aspect [20] of a museum visit be replaced.^23^

Does this mean that virtual museums have become a seasonal phenomenon due to the pandemic, merely a trend that very few will soon want to follow? Or, since the recent events allowed for much broader access to museum collections than ever before, might digitisation be seen as an accelerator of change for more democratised cultural institutions in the future? Will the obstacles virtual museums face be addressed by decision-makers in an effort to promote more democratic and equal societies?

Those questions are left unanswered, at least for now.

## Footnotes

1 The General Assembly of International Council of Museums (ICOM) just recently has approved the definition of museum which states that (among others) a museum is a not-for-profit (…) institution in the service of society (…) for education, enjoyment, reflection and knowledge sharing.

2 Virtual Multimodal Museum (ViMM) is a high-visibility and participative Coordination and Support Action (CSA), funded under the EU Horizon 2020 programme (CULT-COOP-8-2016).

3 Unless otherwise stated, copyright considerations also apply to related rights. Due to the focus of the authors’ article on the aspects of museums’ activities, the general principles of copyright law are mentioned in general terms and only for the sake of completeness.

4 See Article 6bis of Berne Convention for the Protection of Literary and Artistic Works.

5 See Art. 7 of Berne Convention for the Protection of Literary and Artistic Works.

6 “It shall be a matter for legislation in the countries of the Union to permit the reproduction of such works in certain special cases, provided that such reproduction does not conflict with a normal exploitation of the work and does not unreasonably prejudice the legitimate interests of the author”.

7 E.g. „[…] possible inclusion in the scope of application of this Directive of publishers and of works or other protected subject-matter not currently included in its scope, and in particular stand-alone photographs and other images.” In such a situation, the question of who is the rights holder and on what basis also arises. Generally speaking, “[i]f the designers are employed by the museum, their employment contract will normally specify that the museum owns the copyright in all works created in the course of employment. But if the designer is an external contractor then the relevant contract needs to specify that the museum can freely use all relevant media outputs” [24].

8 In such a situation, the question of who is the rights holder and on what basis also arises. Generally speaking, “[i]f the designers are employed by the museum, their employment contract will normally specify that the museum owns the copyright in all works created in the course of employment. But if the designer is an external contractor then the relevant contract needs to specify that the museum can freely use all relevant media outputs” [27].

9 See Recitals 25–26, 30, 53 of the DSM Directive.

10 ACTA, which is an abbreviation for the Anti-Counterfeiting Trade Agreement, was a multilateral treaty which aim was to establish equivalent provisions in international trade agreements containing rules on anti-counterfeiting. Due to a number of controversies over the agreement (including a confidential negotiation process), as well as citizen protests out of concern for restrictions on freedom of expression, the agreement did not enter into force.

11 The Polish government brought an action at the CJEU on 24 May 2019 concerning Article 17, the filtering of online content and the liability of service providers for possible copyright infringements. In the action, which was registered under No. C-401/19 [41], Poland raised the possibility that the provisions of the DSM Directive violated freedom of expression by introducing mechanisms bearing the characteristics of preventive censorship. Ultimately, the action of the Polish government was dismissed.

12 For example, Polish creators have been lobbying for years for the act that would include fair remuneration and other regulations important from their point of view [42, 43].

13 The status of the implementation or the process leading up to it can be checked on the website: https://www.notion.so/DSM-Directive-Implementation-Tracker-361cfae48e814440b353b32692bba879. At the time of finalising this article, most countries had already implemented the relevant regulations, but a large part of them were after the deadline set by the EU legislator.

14 Rights provided for in Article 5(a) and Article 7(1) of Directive 96/9/EC, Article 2 of Directive 2001/29/EC, Article 4(1)(a) of Directive 2009/24/EC and Article 15(1) of DSM Directive.

15 Of all EU Member States, only the Netherlands has introduced an exception narrower than that provided for in Directive 2001/29/EC, see https://copyrightexceptions.eu/#Art.%205.2(c) (accessed 22.08.2022).

16 R. Markiewicz emphasises that “the work itself ‘as such’ cannot be permanently in the collections of an individual, and only its fixation, even if only digital, may have such status” [44:86]. Works, being intangible property, by their nature cannot be in anyone’s collection and the EU legislator probably had copies of works in mind. The wording used may be regarded as a drafting error, which, however, does not change the content and meaning of the regulation.

17 R. Markiewicz even writes that these provisions are “in fact trying to ‘rescue’ the failures of Directive 2012/28 on orphan works” [44:89].

18 See Recital 33 of the DSM Directive.

19 See art. 10(1) and Art. 8(4) of the DSM Directive.

20 This regulation was not in the initial version of the directive, which was rejected in July 2018 by the European Parliament.

21 Germany, Italy and Spain are among the countries that have had to make significant changes to copyright legislation protecting hitherto unoriginal reproductions of works [40].

22 The differences in experiencing a real work of art and its reproduction, as well as the influence of photography on art, already began to be discussed in the 1920s [75].

23 However, the situation may drastically change soon with technical advancements in the VR technology. But even now there are studies indicating that visitors are more likely to be involved in the learning process in a museum when various types of virtual technology are in use to present collections [76].
